# Membrane vesicles produced by next-generation probiotics from the gut as innovative tools for human health

**DOI:** 10.1080/19490976.2025.2552344

**Published:** 2025-09-01

**Authors:** Laura Abraham, Audrey Raise, Laurent Beney, Pierre Lapaquette, Aurélie Rieu

**Affiliations:** UMR PAM, Université Bourgogne Europe, Institut Agro Dijon, INRAE, Dijon, France

**Keywords:** Extracellular vesicles, postbiotics, live biotherapeutics, *Akkermansia muciniphila*, *Faecalibacterium duncaniae*, Next-Generation Probiotics

## Abstract

Probiotics have long been recognized for their health-promoting properties, primarily through their interaction with the gut microbiota. In recent years, Next-Generation Probiotics (NGPs), including *Akkermansia muciniphila* and *Faecalibacterium duncaniae*, have gained attention due to their potential therapeutic applications. Beyond live bacteria, the concept of postbiotics, defined as non-viable bacterial components with health benefits, has emerged, with membrane vesicles (MVs) representing a promising new class. These nanosized extracellular structures, secreted by both Gram-positive and Gram-negative bacteria, are rich in bioactive molecules such as peptides, lipids, metabolites, and nucleic acids. Membrane vesicles have been shown to mediate intercellular communication, modulate immune responses, and influence gut barrier integrity. Their role in microbiota–host interactions makes them attractive candidates for novel therapeutic strategies, particularly in metabolic and inflammatory diseases. This review explores the current state of knowledge on MVs from NGPs, their functional properties, and their impact on health while also discussing future research directions and large-scale production challenges.

## 1. Introduction

In recent decades, the field of probiotics has exploded as a result of major advances in knowledge on the gut microbiota and its crucial role in human health throughout all stages of life, from birth to old age. According to the definition of the FAO/WHO, probiotics are “live microorganisms that, when administered in adequate amounts, confer a health benefit to the host.”^1^ They are most often bacteria from specific genera of proven safety with a long history of use, such as Lactobacilli-related genera or *Bifidobacterium*, but may also include yeasts, such as species from the *Saccharomyces* genus.^2^ Probiotics are thought to be effective in relieving digestive discomforts (e.g., bloating, stomach pain) such as those resulting from irritable bowel syndrome (IBS) and have also been associated with a lower incidence of antibiotic-associated diarrhea^3–5^. Probiotics can protect against both bacterial and fungal pathogens through various mechanisms, including competitive exclusion, the enhancement of mucosal barrier function, immunomodulation, and the production of antimicrobial compounds^6–9^. However, despite promising results in animal models that mimic human diseases, the ability of commercialized probiotics to improve symptoms and/or to prevent the recurrence of specific complex diseases, such as inflammatory bowel diseases (IBDs), is hard to demonstrate statistically and varies significantly between individuals.^10,11^ To be fully effective, orally administered probiotic bacteria have to cope with the stresses of the gastrointestinal tract and establish themselves there, competing with the resident microbiota. One way of potentiating the effect of probiotic bacteria is to grow them in biofilm form, a lifestyle that enhance their resilience to environmental stressors.^12–14^ For instance, *Lacticaseibacillus paracasei* encapsulated biofilms have shown particularly promising properties in terms of resistance to acidic, osmotic and gastrointestinal-related stress, allowing the targeted delivery of this bacteria in the colon.^14^ Beyond the resistance potential of strains used as probiotics, the fact that bacteria currently used as probiotics do not represent major inhabitants of a healthy human gut microbiota, or are not naturally commensal, can be another explanation of their lack of efficiency and their difficulty in achieving the permanent colonization of the gut^15^. Thus, investigating the probiotic potential of the key species within the gut microbiota is of great interest. In this context, the conventional definition of probiotics has evolved alongside scientific discoveries to include Next-Generation Probiotics (NGPs). Unlike classical probiotics, the latter do not have an established history of safe use in humans. Instead, NGPs are directly isolated from the human microbiota and then selected for their biological effects and potential therapeutic activities.^16^

**Figure 1.**
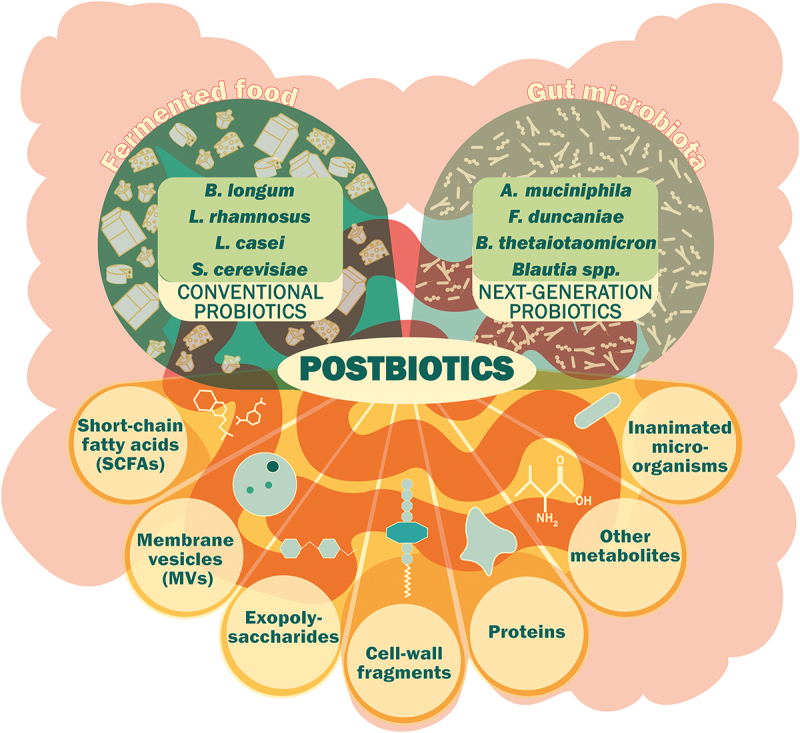
Overview of conventional probiotics and next-generation probiotics along with their derived postbiotics.

The traditional definition of probiotics emphasizes the administration of living organisms. However, the beneficial effects of some probiotic strains do not necessarily require them to be alive and, in some cases, their isolated metabolites can recapitulate these effects. This is well illustrated by the preservation of the beneficial metabolic effects on a host of a pasteurized form of *Akkermansia muciniphila* and the beneficial effects obtained with a purified membrane protein of this bacterium.^17^ This has led to the emergence of the concept of postbiotics. Postbiotics are defined as the “preparation of inanimate microorganisms and/or their components that confers a health benefit on the host” and this definition is currently used by the International Scientific Association for Probiotics and Prebiotics (ISAPP).^18^ Postbiotics exclude viruses (as well as bacteriophages), vaccines, filtrates without cell components, purified microbial components (proteins, peptides, exopolysaccharides) and purified microbial metabolites (e.g., organic acids).^18^ The current definition of postbiotics is a topic of ongoing debate. Salminen *et al*., include inactivated microorganisms along with their specific products and components, which has caused confusion among scientists. The concern arises from the inability to clearly distinguish well-defined molecular factors from more complex or undefined mixtures derived from microbial cells, leading to ambiguity in the classification and understanding of postbiotics.^19^ A summary figure, illustrating the nuance between conventional probiotics and NGPs as well as various examples of postbiotics, is shown in Figure 1.

Among microorganism-derived compounds, membrane vesicles (MVs) are nanoscale particles composed of a lipid bilayer, released by various microbes, and are emerging as promising candidates for postbiotics. MVs are released by species across the three domains of life (Archaea, Bacteria and Eukarya), highlighting the evolutionary conservation and fundamental importance of the vesiculation process.^20^ MVs consist of a lipid bilayer encapsulating and harboring a variety of components, including proteins, nucleic acids, toxins, and more, all originating from the parent bacterium’s content.^21^ Although the content of MVs generally reflects that of the originating cell, it can be selectively enriched with specific molecules.^22^ MVs participate in a variety of important physiological processes in bacteria, such as inter- or intra-species communication, interaction with the host, biofilm development, phage defense, horizontal gene transfer, and virulence in pathogenic bacteria.^23^ In recent years, MVs have gained relevance in the health sector due to their ability to act as long-distance transporters and are being explored as novel therapeutic agents. For instance, bacterial MVs have been suggested to reach the host brain from the gut compartment.^24^

Despite ongoing debates surrounding the definition of postbiotics, MVs align with the various classifications proposed. This is because MVs represent non-viable cell components that carry bioactive metabolites, distinguishing them from live probiotics while still delivering therapeutic potential. Their ability to maintain biological activity without the presence of live bacterial cells strengthens their position as a valuable tool in postbiotic-based therapies. Of note, microbial MVs are also being explored as innovative carriers for encapsulating synthetic active principles, including drugs, vaccines, antimicrobial agents, and nucleic acids.^25^

This review aims to compile the current knowledge of MVs released by the main NGPs and their potential as biotherapeutic agents, providing examples of several studies on MV activity and discussing the putative molecular mechanisms involved in these effects.

## 2. Next-generation probiotics: health allies from the gut

The development of advanced sequencing methods and cultivation techniques has accelerated the discovery rate of novel bacteria with a probiotic potential.^26,27^ These bacteria originate from the gut microbiota and are classified as NGPs, as they belong to bacterial genera that have never been used as probiotics, or in the agri-food industry. Many bacterial strains used as NGPs belong to the Actinobacteria, Bacteroides, Firmicutes, and Verrucomicrobia phyla.^28^ Since the gut lumen is deprived of oxygen, most of these bacteria are strictly anaerobic or even extremely sensitive to oxygen. One difference between probiotics and NGPs is that the latter are more susceptible to follow a drug delivery framework.^26^ Indeed, NGPs also fit the Living Biotherapeutic Products definition which includes three criteria, they: “(1) contain live organisms, such as bacteria; (2) are applicable for the prevention, treatment, or cure of a disease or a condition in human beings; and (3) they are not a vaccine”^26^. Unlike usual probiotics, NGPs undergo clinical trials (phases I – IV) and must be approved by relevant regulatory authorities before being put on the market.^26^

To guarantee the safety of the strains, it is necessary to ensure that they do not present any undesirable effects for humans. Comprehensive evaluations should include *in vitro* analyses of bacterial physiology, genomic assessments to detect potential virulence factors and the identification of genes associated with the transfer of antibiotic resistance. Additionally, *in vivo* acute toxicity studies should be conducted in both healthy and immunosuppressed mice to ensure the strain’s safety across different physiological conditions.^28^ It is essential to preserve cell viability and functionality throughout the entire production process, from biomass cultivation and downstream stabilization to protective formulation and final administration to the patient. For gut commensal bacteria, in particular, it is essential to protect the microbial cells from exposure to oxygen, moisture, and temperature fluctuations during production and storage. Additionally, the bacteria, in their oral administration formula, must be able to withstand the acidic pH of the stomach, digestive enzymes, and bile salts encountered during transit in the upper part of the gastrointestinal tract^29^. It is also important to assess whether the repeated administration of such microorganisms could negatively impact the composition and functions of the resident gut microbiota. Unlike traditional probiotics, which are designed for a broad target population and primarily focus on gut health, NGPs are being developed with a more precise focus on addressing specific diseases, including those affecting organs distant from the gut.^28^ Moreover, we can hypothesize that the administration of these NGPs may have the most pronounced beneficial effects in patients who exhibit a marked reduction in the abundance of these bacteria, thereby restoring their representation. For instance, only a subset of Crohn’s disease patients showed a decrease in the NGP-potential bacterium *Faecalibacterium duncaniae*.^30^ It is worth considering that the beneficial effects of NGPs, likewise their mode of action, are specific to the bacterial strain considered. Examples of bacteria with high potential as NGPs are given in Table 1, along with the diseases they are associated with or could potentially prevent or treat.

**Table 1.** Example of bacteria from the new generation of probiotics and their main target diseases.

Bacterial species/strain	Targeted diseases	Metabolism	References
*Anaerobutyricum soehngenii spp.*	Metabolic diseases	Strict anaerobe	^31^
*Akkermansia muciniphila* Muc^T^	Obesity, metabolic diseases, IBD	Strict anaerobe	^32,33^
*Bacteroides fragilis* ZY-312	Pathogen infection, ulcerative colitis	Aerotolerant	^34–36^
*Bacteroides dorei* D8	Cardiovascular diseases	Anaerobe	^37,38^
*Bacteroides thetaiotaomicron* DSM 2079	IBD	Strict anaerobe	^39,40^
*Blautia spp.*	Obsesity, diabetes, inflammatory diseases	Strict anaerobe	^41–43^
*Christensenella minuta* DSM 22,607	IBD	Oxygen-tolerant	^44,45^
*Clostridium butyricum* CBM 588	Colorectal cancer, Inflammatory diseases, pathogen infection	Strict anaerobe	^46–49^
*Faecalibacterium duncaniae*	IBD, IBS, respiratory infection, diabetes	Strict anaerobe, EOS	^30,50–52^
*Roseburia intestinalis spp.*	Cardiovascular diseases, Crohn’s disease,	Strict anaerobe	^53,54^

Abbreviations: EOS, extremely oxygen sensitive; IBD, intestinal bowel disease: IBS, irritable bowel syndrome.

Among the bacterial strains listed in Table 1, two are gaining attention as promising candidates as NGPs due to their high therapeutic potential: *Akkermansia muciniphila* Muc^T^ and *Faecalibacterium duncaniae* (formerly known as *F. prausnitzii* A2–165)^55^. Although several next-generation probiotic candidates are currently being characterized and/or developed for commercial applications, this review will focus primarily on *Akkermansia muciniphila* and *Faecalibacterium duncaniae*. This selection is driven by the extensive literature available on these two organisms, as well as their advanced stage of translational development. Notably, *A. muciniphila* is already being marketed by The Akkermansia Company, recently acquired by Danone, while *F. duncaniae* is currently undergoing phase 2 clinical evaluation with EXL01, a first-in-class immunomodulator derived from *F*. *prausnitzii* by the start-up Exeliom Biosciences. Furthermore, these microorganisms exhibit different functional profiles: *A. muciniphila* is predominantly associated with beneficial effects in metabolic disorders, whereas *F. duncaniae* is primarily recognized for its potent anti-inflammatory properties in the context of IBDs. Nonetheless, additional promising NGP candidates will also be discussed throughout this review.

### 2.1. Akkermansia muciniphila

This strictly anaerobic Gram-negative bacterium was isolated for the first time in 2004.^32^ It is one of the most abundant bacteria in the gut microbiota, with a relative abundance estimated between 0.5% and 5% of total gut bacteria.^32,56^ *Akkermansia muciniphila* resides in the mucus layer lining the digestive tract, where it degrades mucin, and has been linked to the prevention and improvement of obesity and other metabolic disorders^32^. A reduction in its prevalence has been observed in individuals with obesity and type 2 diabetes, suggesting a possible causal link with the onset of such metabolic disorders.^57,58^ It was thought that the beneficial effects of microorganisms used as food supplements would disappear when the bacteria were inactivated (e.g., pasteurization). However, results have shown that pasteurized bacteria can not only retain their beneficial effects but, in the case of *A. muciniphila*, may even enhance them.^17^ *A*. *muciniphila* seems to exert its effects on the host mainly through the release of outer membrane proteins (e.g., the pili-like proteins Amuc_1100 and the P9 protein Amuc_1631), metabolites (e.g., harmaline) including short chain fatty acids (SCFAs; e.g., propionate and acetate) and cell envelope compounds (diacyl phosphatidylethanolamine and peptidoglycan-derived muropeptides).^59^ These compounds can act on multiple key host signaling pathways that regulate metabolism and immunity, such as the Toll-like receptors (TLRs)/Nucleotide Oligomerization Domain-like receptors (NLRs)-associated signaling, the secretion of the glucagon-like peptide-1 (GLP1) involved in satiety and glucose metabolism, or the Takeda G protein-coupled receptor 5 (TGR5) signaling.^59^ In the literature, the administration of this bacteria in mice models has been shown to reduce food intake, body weight, and insulin resistance while increasing the gut barrier function^60,61^. In a clinical study carried out on 32 volunteers suffering from metabolic diseases such as overweight, obesity and insulin resistance, the pasteurized form of this bacterium was more effective than the probiotic form in treating these pathologies.^62^ The administration of this bacterium was well-tolerated without reported adverse effects. As a result, the safety of the pasteurized form of *A. muciniphila* was recently validated by the EFSA, authorizing its commercialization as a novel food in Europe.^63^ However, a recent study by Gleeson et *al*. has raised concerns about excessive levels of *A. muciniphila* and its potential role in autoimmune nephropathy. The study found that the bacterium degrades sugars on immunoglobulin A (IgA) in the intestinal mucus, and these deglycosylated immunoglobulins can reenter the bloodstream, where they are mistaken as foreign by the immune system.^64^ This triggers antibody sequestration, leading to IgA accumulation in the kidneys and resulting in renal insufficiency.^64^ Thus, both a deficiency and an overabundance of *A. muciniphila* in the gut might be detrimental and associated with disease development.

The use of MVs might limit the harmful effects described above that can be associated with administering viable bacteria to the host. In addition, MVs enable the encapsulation and protection of bioactive cargo, allowing their transport and distribution to peripheral organs. In mice, orally administered fluorescent outer membrane vesicles (OMVs) were shown to gradually disseminate from the gastrointestinal tract to distant tissues, with sustained signals detected in digestive organs and notable accumulation in the skin over several days.^65^

### 2.2. Faecalibacterium duncaniae

This bacterium belongs to the *Faecalibacterium* genus, which is a genus of strictly anaerobic, extremely oxygen-sensitive, Gram-positive, rod-shaped, non-motile bacteria.^50^ At present, this bacterium is classified as Gram-positive, although several studies have reported that it stains Gram-negative and Gram-variable.^66,67^ One hypothesis suggests that this discrepancy arises from its unique cell wall composition: unlike typical Gram-positive bacteria, this species may have a thin layer of peptidoglycan, which could affect the staining outcome. If *F. duncaniae* were Gram-negative, it would be expected to activate TLR4 through its ligand LPS, a component commonly found in the outer membrane of traditional Gram-negative bacteria.^66^ However, *in silico* analysis showed no TLR4 activation by either live or UV-killed bacteria, suggesting the absence of LPS in this species, and therefore arguing against the hypothesis that this bacterium is Gram-negative.^66^ Further analyses are now expected to determine *F. duncaniae’s* cell envelope structure and to which Gram it belongs. For the remainder of this review, *F*. *duncaniae* will be considered Gram-positive, as indicated by its current classification.

*F*. *duncaniae* accounts for an average of 6.5% of the human gut microbiota, positioning it among the most abundant bacterial genera detected in the intestinal ecosystem.^68^ The interest of this bacterium was initially revealed by its lower abundance in the ileal mucosa of Crohn’s disease patients with endoscopic recurrence, along with *in vitro* and *in vivo* studies in animal models, demonstrating its anti-inflammatory properties.^69^ *F. duncaniae* is an acetate-consuming bacterium that secretes metabolites like SCFAs such as butyrate which favorably modulates the intestinal immune system, oxidative stress, and colonocyte metabolism.^70,71^ In addition, *F. duncaniae* has been shown to secrete anti-inflammatory compounds such as salicylic and shikimic acids to its surrounding environment.^72^ In addition, *F. duncaniae* is a strong producer of a microbial anti-inflammatory molecule (MAM) with anti-inflammatory properties that are thought to attenuate colitis *in vivo* and decrease the activation of nuclear factor-ĸB (NF-κB) signaling.^73^ People suffering from IBDs present a lower number of *F. duncaniae* in their feces compared to healthy people.^74^ These differences in *F. duncaniae* abundance could be used as potential biomarkers and help to detect IBDs.^74^

Despite the positive aspects described before, NGPs do have some limitations. Indeed, as mentioned before, the definition of such products relies on the use of living microorganisms. As NGP strains have not previously undergone large-scale and long-term use, their potential to cause disease is uncertain. What is more, their extreme sensitivity to oxygen makes these strains difficult to cultivate at the laboratory scale and represents a barrier to their industrial-scale cultivation and stabilization, despite the promising NGP formulation strategies developed at the lab scale.^26,75^ Indeed, lack of knowledge and techniques complicates these various stages and prevents them from being marketed. As a result, interest is now focusing on the postbiotics derived from these NGPs, such as MVs.

### 2.3. Anaerobutyricum soehngenii

*Anaerobutyricum soehngenii* (*A. soehngenii*) is a Gram-positive, strictly anaerobic bacterium that was first isolated from the feces of healthy infants in the 1990s.^76^ It is a commensal member of the human gastrointestinal microbiota and a prominent producer of butyrate. Unlike the well-characterized sugar-fermenting butyrate producers *F. duncaniae* and *Roseburia intestinalis*, *A. soehngenii* preferentially metabolizes both D- and L-lactate in the presence of acetate to generate butyrate.^77^ Interest in this species has grown following fecal microbiota transplantation studies that identified it as a candidate health-promoting microbe. Oral administration of *A. soehngenii* has been reported to improve peripheral insulin sensitivity, elevate glucagon-like peptide-1 (GLP-1) secretion, and reduce glycemic variability, underscoring its therapeutic potential in metabolic disorders^31^.

### 2.4. Bacteroides fragilis

*Bacteroides fragilis* (*B. fragilis*) is a Gram-negative, aerotolerant anaerobe predominantly found in the human colon^35^ This species exists in two distinct forms: a pathogenic strain capable of producing enterotoxins, referred to as enterotoxigenic *B. fragilis* (ETBF), and a non-toxigenic strain (NTBF), which has been associated with probiotic properties. *B. fragilis* displays extensive metabolic versatility, notably through its capacity to degrade complex polysaccharides. Genes involved in polysaccharide utilization have been linked to modulation of the gut microbiota composition and enhanced production of SCFAs, both of which are critical for host metabolic and immune homeostasis. Administration of the non-toxigenic *strain B. fragilis* ATCC 25,285, as well as its culture supernatant, has demonstrated anti-inflammatory effects and significantly attenuates dextran sulfate sodium (DSS)-induced colitis in murine models.^78^ The strain *B. fragilis* ZY-312 also demonstrates a protective effect to the host against the pathogen *Cronobacter sakazakii*.^35^

### 2.5. Bacteroides dorei

*Bacteroides dorei* (*B. dorei*) is a Gram-negative, strictly anaerobic rod-shaped bacterium that is predominantly found in the human colon.^37^ A specific strain, *B. dorei* D8, isolated from a fresh human stool sample, has demonstrated the ability to reduce luminal cholesterol levels. This cholesterol-lowering activity suggests a potential role for *B. dorei* D8 in mitigating the risk of cardiovascular disease through the modulation of host lipid metabolism.^38^

### 2.6. Bacteroides thetaiotaomicron

*Bacteroides thetaiotaomicron (B. thetaiotaomicron*) is a Gram-negative, strictly anaerobic bacterium that resides in the human gastrointestinal tract.^39^ This species has been associated with several beneficial effects on host health, including the enhancement of the intestinal mucosal barrier and inhibition of pathogenic invasion.^79^ Moreover, *B. thetaiotaomicron* exhibits anti-inflammatory properties, as evidenced by its ability to downregulate pro-inflammatory gene expression in a murine model of DSS-induced colitis.^40^ In addition, *B. thetaiotaomicron* appears to exhibit a favorable safety profile and is well tolerated in patients with Crohn’s disease.^80^

### 2.7. Blautia spp

Blautia spp. are strictly anaerobic bacteria that form an integral part of the human gut microbiota.^41^ All species within this genus are Gram-positive, with the exception of *Blautia massiliensis*, which is Gram-negative.^81^ Among these, *Blautia wexlerae* has been shown to exert anti-inflammatory effects and modulate the intestinal environment in a host-beneficial manner.^42^ To elucidate the underlying mechanisms, comprehensive analyses of its amino acid and carbohydrate metabolic pathways were conducted, revealing bioactive metabolites likely acting synergistically to attenuate metabolic disorders, including obesity and type 2 diabetes.^42^ Furthermore, administration of *Blautia faecis* DSM 33,383 was found to significantly reduce the severity of post-influenza bacterial superinfection caused by *Streptococcus pneumoniae* in the lungs, suggesting a broader immunomodulatory potential of *Blautia* species.^43^

### 2.8. Christensenella minuta

*Christensenella minuta* (*C. minuta*), a member of the Christensenellaceae family, is a subdominant Gram-negative commensal bacterium within the human gut microbiota.^44,118^ Although classified as a strict anaerobe, *C. minuta* displays an unusual tolerance to oxygen, which may contribute to its resilience within the fluctuating intestinal environment.^44^ Similar to *F. duncaniae*, its relative abundance is markedly reduced in individuals with IBDs. This species has demonstrated strong anti-inflammatory activity, notably through the suppression of interleukin-8 (IL-8) production *via* the inhibition of the NF-κB signaling pathway.^44^ In addition, *C. minuta* has been shown to protect against intestinal tissue damage and reduce colonic inflammation in preclinical models.^44^ These properties position *C. minuta* as a promising NGP candidate for the prevention and treatment of IBDs.

### 2.9. Clostridium butyricum

*Clostridium butyricum* (*C. butyricum*) is a Gram-positive, strictly anaerobic, spore-forming bacterium.^46^ Initially isolated from the intestinal tract of pigs, *C. butyricum* is now recognized as common commensal species in both human and animal gastrointestinal microbiota. It has also been isolated from a wide range of environmental sources, including soil, vegetables, fermented dairy products such as soured milk, and cheese.^46^ In addition, its ecological versatility, *C. butyricum* has demonstrated significant immunomodulatory potential. Recent studies have shown that it enhances the efficacy of colorectal cancer immunotherapy by promoting CD8^+^ and CD4^+^ T cell infiltration into tumor sites.^82^ In addition, this bacterium was shown to provide anti-inflammatory properties by preventing acute experimental colitis in mice through the induction of the immunoregulatory cytokine IL-10 and to limit the colonization of pathogenic bacteria in the gut.^47,83^

### 2.10. Roseburia intestinalis

*Roseburia intestinalis* (*R. intestinalis*) is a strictly anaerobic Gram-positive bacterium that was first isolated from human fecal samples.^84,85^ It is a prominent member of the gut microbiota known for its ability to produce butyrate. Preclinical studies suggest that it may exert anti-inflammatory effects, in particular by promoting the differentiation of regulatory T cells (Tregs), a mechanism potentially involved in the modulation of intestinal inflammation, although its relative abundance appears reduced in patients with IBD, including Crohn’s disease.^85^ In addition to its immunomodulatory properties, this species has demonstrated a protective role against atherosclerosis, an effect that is likely mediated by its butyrate production and its influence on host lipid metabolism and systemic inflammation.^53^

## 3. Membrane vesicles by NGP: a new class of postbiotic

Emerging studies have shown that MVs from gut microbiota play a crucial role in microbiota host-interaction. Membrane vesicles, also known under the generic term of extracellular vesicles (EVs), are non-viable nanoparticles with diameters ranging from approximately 20 to 400 nm, released by cells.^86^ MVs were observed for the first time in *Escherichia coli* in 1960s and have been studied extensively since then.^87^ An increase in MV production has been observed in several stressful conditions, showing that bacterial vesiculation can be associated with a stress-response.^88^ The gut lumen appears to be a suitable environment for the production of MVs by the gut microbiota, as indicated by the isolation of bacterial MVs from human fecal matter.^89,119^

### 3.1. Gram-negative NGPs-MVs

In Gram-negative bacteria, MVs are referred to as OMVs because their lipid composition resembles that of the bacterial outer membrane from which they primarily originate.^23^ These vesicles can be formed in two main ways: either through blebbing of the outer membrane or via explosive cell lysis.^86^ Some Gram-negative NGP-MVs have already been characterized, particularly with regard to the size of the MVs produced. The reported average diameter of the vesicles collected can vary from one study to another. In one study, MVs released by *A. muciniphila* ATCC BAA-835 (MucT type strain) were reported to range in size from 30 to 150 nm using transmission electronic microscopy (TEM), while another study using the same strain found sizes ranging from 30 to 350 nm using nanoparticle tracking analysis (NTA), with an average peak size of 121.6 nm.^90,91^ In contrast, a third study reported an average peak size between 40 and 60 nm for the same strain using dynamic light scattering (DLS).^92^ These differences may arise from variations in culture conditions, purification methods, or characterization protocols. While all three studies employed ultracentrifugation to isolate MVs, differences in culture media and technologies used to measure vesicle size likely contributed to the observed variability, even within the same bacterial strain. Regarding the composition of these vesicles, although the Amuc_1100 protein is associated with the bacterial cell wall, its presence in MVs has not been confirmed in recent studies but remains highly probable.

### 3.2. Gram-positive NGPs-MVs

Although MVs released by Gram-negative bacteria are well documented, Gram-positive bacteria have been neglected in this field due to the belief that their thick cell wall prevents the passage of vesicles.^93^ However, recent publications have shown that MVs are also released by several Gram-positive bacteria.^94^ Since these bacteria do not have an outer membrane, these vesicles can be referred to as cytoplasmic membrane vesicles (CMV).^94^ CMV biogenesis in Gram-positive bacteria is more challenging and in most cases requires the action of enzymes such as phage-derived endolysins or stress-induced autolysins, which degrade peptidoglycan and allow the cytoplasmic membrane to form vesicles.^95–98^ Ultimately, these vesicles resemble those produced by Gram-negative bacteria, they consist of a lipid bilayer enriched in specific lipids and proteins originating from the Gram-positive bacteria.

For instance, MVs released by the Gram-positive NGP *F. duncaniae* have sizes ranging from around 20 to 225 nm.^99,100^ However, detailed identification of the protein content of these MVs, including specific proteins, still requires in-depth proteomics work.

**Figure 2.**
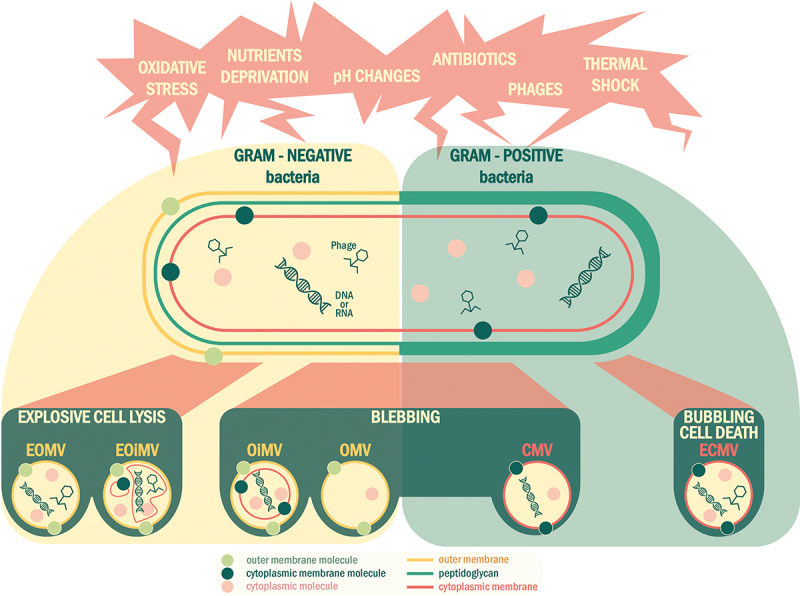
Various environmental stresses induce the formation of bacterial membrane vesicles (MVs). The biogenesis of MVs follows different pathways specific to gram-negative (explosive cell lysis and blebbing) and gram-positive bacteria (blebbing and bubbling cell death). Abbreviations: EOMV, explosive outer membrane vesicles; EOIMV, explosive outer-inner membrane vesicles; OIMV, outer-inner membrane vesicles; OMV, outer membrane vesicles; CMV, cytoplasmic membrane vesicles; ECMV, explosive cytoplasmic membrane vesicles.

Although the secretion of MVs is part of the basal bacterial metabolism, as mentioned above, their production can be higher under stressful conditions to help bacteria adapt (Figure 2). For instance, oxidative stress, nutrient deprivations, pH changes, and antibiotics can induce vesiculation in bacteria^88^. Thus, the environment plays an important role in the induction of MV biogenesis and composition, as do bacterial type and the biogenesis pathway involved.^86^ Of note, the production of MVs by bacteria has been proposed as a new secretion system: secretion system type 0 (SST0).^101^ Further studies are now necessary to decipher the precise molecular mechanisms by which NGPs can produce MVs, and how environmental factors can modulate this production quantitatively and qualitatively (i.e., MV content). Since it is known that the presence of environmental stresses such as the presence of antibiotics, envelop stress or DNA damage stimulates MVs biogenesis, vesiculation might be associated with a biological stress response or even with cell death.

### 3.3. Isolation and characterization of NGPs-MVs

The isolation of MVs from cells and other compounds in the bacterial culture supernatant is a critical step in vesicle research. Proper separation is essential to obtain reliable results and prevent misinterpretations due to the presence of contaminating nanoparticles, such as soluble proteins. MV isolation can be based on size, density, or immunophenotype.^102^ For size-based separation, methods such as differential centrifugation, size-exclusion chromatography, and ultrafiltration are commonly employed. Density-based isolation can be performed using density gradient centrifugation or chemical precipitation. Alternatively, immunophenotype-based methods involve immunocapture assays using monoclonal antibodies targeting specific surface markers. To enhance both the purity and yield of MVs, it is often beneficial to combine several techniques.

Once isolated, the MVs must be characterized in terms of size, morphology, and composition. Bacterial MVs are generally in the same size-range as eukaryotic exosomes (30–120 nm), but smaller than microvesicles (150–1000 nm) and apoptotic bodies (100–5000 nm), which are other types of vesicles produced by eukaryotic cells. Research on eukaryotic vesicles is much more advanced than that on bacterial MVs, and common characterization tools such as NTA, DLS, and VideoDrop may lack sufficient resolution to accurately assess bacterial MV size. Microscopy techniques provide valuable structure insights: TEM is widely used to determine MV size and morphology with high resolution, while atomic force microscopy (AFM) offers complementary three-dimensional surface analysis at nanometric precision. Size alone does not allow the complete identification of vesicles, and the use of specific biomarkers is essential. Unlike eukaryotic extracellular vesicles, bacterial MVs biomarkers are still poorly characterized. For each NGP, identifying genus- or species-specific markers is necessary to ensure precise characterization and isolation. In this context, published lipidomic and proteomic datasets are valuable for the discovery and validation of such markers. Despite current limitations, it is possible to distinguish MVs from Gram-negative and Gram-positive bacteria, primarily due to differences in their cell wall structure, which influences the lipid and protein content of the vesicles. For instance, OMVs from Gram-negative bacteria contain lipopolysaccharides (LPS), whereas CMVs from Gram-positive bacteria may contain lipoteichoic acids.^103^

## 4. Biomedical applications of MVs released by NGPs

**Figure 3.**
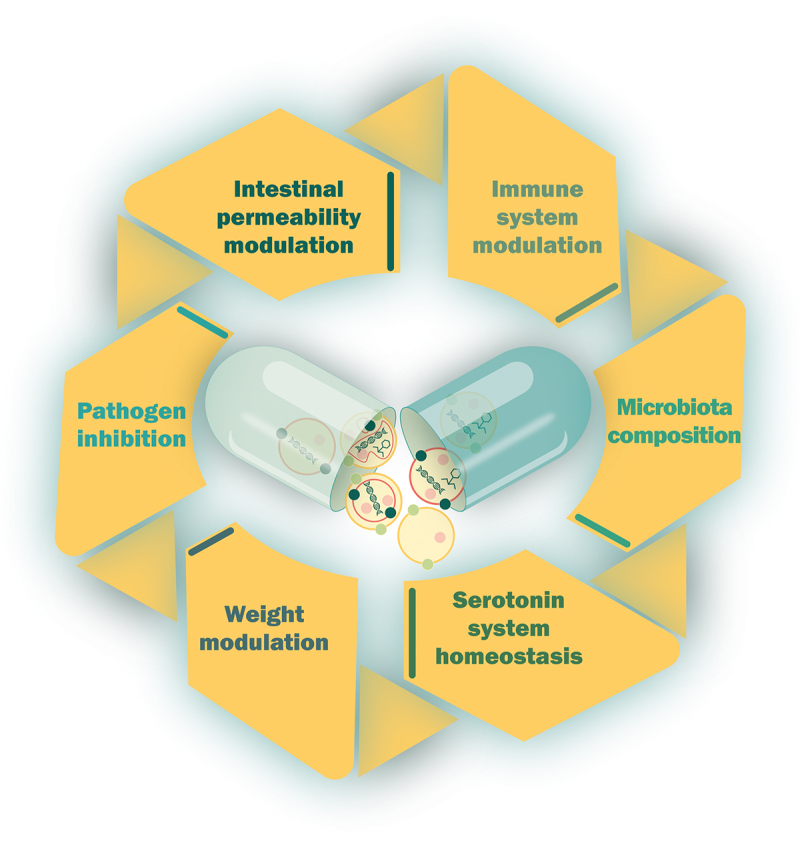
Human health applications of membrane vesicles from next-generation probiotics.

Membrane vesicles are key players in various physiological and pathological processes, as they can transport biologically active macromolecules from one microbial cell to another, and at distance, to host cells throughout the body. Indeed, bacterial MVs produced in the gut have been shown to diffuse across various host tissues, including the liver, heart, spleen, kidneys, and even the brain^104^, significantly influencing the functions of target cells (Figure 3).^105^ This unique ability positions them as promising agents for diverse therapeutic applications. Moreover, it has been noticed that MVs released by bacteria can interact with host cell surface receptors but can also be internalized into host cells through several routes including phagocytosis, clathrin- or caveolin-mediated endocytosis, macropinocytosis, and lipid rafts.^106–108^

### 4.1. Intestinal bowel diseases (IBDs)

A study by Zheng *et al*., revealed that MVs produced by *A. muciniphila* ATCC BAA-835 prevented colitis symptoms and reduced colonic tissue injury in mice. They demonstrate that these MVs exhibited an anti-inflammatory activity in LPS-stimulated RAW 264.7 macrophages, and that these MVs could be internalized by the macrophages.^91^ They also investigated *A. muciniphila* MVs *in vivo* by oral gavage of these MVs for 3 weeks in mice. Their results showed that the administration of these MVs significantly inhibited weight loss and colon shortening in DSS-induced colitis in mice. This supplementation with MVs also decreased pro-inflammatory factors, improved intestinal permeability, and increased the expression of tight-junction genes.^91^ Another interesting parameter was highlighted in this study since MVs were shown to be sufficient to regulate the intestinal microbiota balance by elevating the quantity of *Firmicutes* and reducing the quantity of *Proteobacteria*, counterbalancing colitis-induced dysbiosis.^91^

More recently, *in vivo* administration of MVs from *F. duncaniae* has been shown to attenuate DSS-induced colitis in mice by modulating the intestinal mucosal barrier by increasing zonula occludens-1 (ZO-1) and occludin protein levels and increasing the ratio of CD4^+^ CD25^+^ FOXP3^+^ Tregs in colonic tissue.^109^ Further research is still required to identify the specific components of MVs responsible for mediating the observed intestinal anti-inflammatory effects. In addition, MVs from *F. duncaniae* may be efficient in alleviating symptoms of IBDs. Results at the transcriptional level showed that *F. duncaniae-*derived MVs can modulate the permeability of a human intestinal epithelial cell monolayer (Caco-2) *in vitro* through the upregulation of genes encoding tight junction proteins (occludin and ZO-1) and genes encoding the nuclear receptors of the PPAR (peroxisome proliferator-activated receptor) family.^99^

MVs produced by *Roseburia intestinalis* were shown to provide a positive impact on intestinal recovery and gut microbiome alteration in treating IBDs.^110^ After oral administration, MVs were accumulated in inflamed colonic tissues and increased the abundance of *Bifidobacterium*, reducing colonic inflammation and supporting intestinal healing in mice.^110^ Due to the presence of Ile-Pro-Ile in the vesicular structure, these MVs produced by *R. intestinalis* KCTC 15,746 reduced DPP4 activity in inflamed colonic tissue and increased active GLP-1, thereby downregulating NF-κB and STAT3 via the PI3K signaling pathway and, by doing so, reducing inflammation.^110^

### 4.2. Metabolic disease

The oral administration of *A. muciniphila* ATCC BA-835 was shown to improve gut permeability in high fat diet (HFD)-fed mice by regulating tight junctions through AMP-activated protein kinase (AMPK) activation.^92^ The administration of these MVs improves the glucose tolerance in HFD-induced diabetic mice. In addition, mice fed with a HFD showed reduced colon length, which was improved following oral gavage with MVs. They also observed that these MVs were able to penetrate the large intestine and spread to peripheral tissues (fat tissue, liver, and muscle).^92^

### 4.3. Gut homeostasis

In a study focusing on serotonin system-related genes in human Caco-2 cells, Yaghoubfar *et al*., showed that *A. muciniphila* ATCC BAA-835 and *F. duncaniae*-derived MVs could impact the expression of major genes involved in the serotonin system.^100^ Further studies are now necessary to explore whether these NGPs-derived MVs can be useful *in vivo* to maintain the homeostasis of the serotonin system. Recent work suggests that serotonin plays a key role in modulating immune responses, gut microbiota and autophagy, a key cellular process for maintaining homeostasis.^111^

In addition, MVs released by *B. thetaiotaomicron* VPI-5482 can play a crucial role in the degradation of complex polysaccharides, contributing to host nutrition and the maintenance of intestinal homeostasis.^112^ In addition, these vesicles can modulate immune responses by carrying bioactive molecules, potentially influencing the balance between tolerance and inflammation in the gut.^112^ However, further research is needed to fully understand the impact of MVs released by *B. thetaiotaomicron* on human health and their therapeutic potential.

### 4.4. Virulence modulation

MVs released by *B. thetaiotaomicron* VPI-5482 were shown to inhibit virulence gene expression from the enteric pathogen *Shigella flexneri*.^113^ In their study, Xerri and Payne demonstrated that this effect is attributed to the lipids composing the vesicles and is not related to the presence of a cargo encapsulated in MVs.^113^

### 4.5. Host immunity

MVs released by *Bacteroides fragilis*, another NGP candidate, were investigated. These MVs were shown to have an immunomodulatory effect at the mucosal surface.^114^ Indeed, *B. fragilis* NCTC 9343 MVs were shown to activate the single-stranded RNA receptors TLR7 in HEK-Blue cells while alive and heat-killed *B. fragilis* could not. This activation is associated with the presence of RNA cargo inside MVs which is directly transported into the host epithelial cells to activate host PRRs such as TLR7.^114^

### 4.6. Pre-eclampsia

In another study, gut microbiota dysbiosis was associated with pre-eclampsia, which is a pregnancy-specific syndrome.^115^ The protective role of *A*. *muciniphila* ATCC BAA-835 against pre-eclampsia was mainly associated with MVs secretion. The vesicles traveled from the gastrointestinal tract into the placenta and interacted with trophoblasts, ameliorating pre-eclampsia phenotypes in a murine model.^115^

## 5. Conclusion and challenges

Given the extreme sensitivity of NGPs to oxygen, large-scale production remains a critical objective for their therapeutic application. In this context, the use of MVs produced by these bacteria represents a promising strategy to overcome technological limitations. Compared to live bacteria, MVs offer greater stability and biocompatibility, making them promising candidates as postbiotics for treating specific diseases. To enhance research in this field, it is essential to identify the bioactive compounds within the vesicular fraction that are responsible for their therapeutic effects. Depending on the purification protocols, the vesicular fraction can contain various compounds co-purified with MVs, it is therefore important to be cautious about conclusions regarding effects that can be specifically and solely attributed to MVs. It would also be interesting to investigate whether a complex mixture of vesicles and components from the supernatant such as proteins or fatty acids (e.g., butyrate) could enhance the bioactivity of the vesicular fraction. However, most studies demonstrating the bioactivity of MVs have been conducted at the laboratory scale. A key challenge lies in the design of industrial processes and, more specifically, in the optimization of the separation and purification operations required for the standardization and quality assessment of the products. Recent technological advances have facilitated the development of relatively large-scale anaerobic bioreactor systems, enabling NGPs to be cultivated at a pre-industrial scale under controlled laboratory conditions. These advances have significantly increased the yield of MVs available for downstream applications. However, the methods commonly used for MV isolation in research settings are often difficult to scale up and are not readily compatible with industrial production requirements. Among the emerging scalable approaches, tangential flow filtration (TFF) appears particularly promising for the concentration and purification of NGP-derived MVs, due to its efficiency, reproducibility, and potential for integration into large-scale manufacturing workflows.^116^ The large‑scale production of MVs and their delivery for applications require storage conditions that preserve vesicle integrity, bioactivity, and particle concentration. Short‑term preservation (≤1 week) is acceptable at 4°C, but long‑term storage should be maintained at −80°C, which retains particle counts, nucleic‑acid cargo, morphology, and functional activity more effectively than at − 20°C^117^. In addition, repeated freeze – thaw cycles should be avoided because they decrease MV yield, increase the size, and aggregate particles, and diminish biological activity.^117^ Emerging strategies such as lyophilization and hydrogel encapsulation have shown promise in preserving MVs, by enhancing their stability and maintaining bioactivity during storage, even at room temperature. Lyophilization, particularly when combined with cryoprotectants like trehalose, sucrose, and polysorbate 80, preserves MV morphology, protein content, and functional properties^117^. Hydrogels also offer a stabilizing matrix and enable controlled release, making them attractive for therapeutic applications.^117^ Although Ahmadian et *al*. focused on eukaryotic EVs, the underlying physicochemical principles are likely applicable to bacterial MVs. As research on NGP-derived MVs and their biogenesis continues to develop, it is expected that technical barriers will be progressively overcome, paving the way for MVs to become a viable alternative to current biotherapies.
